# Conversion in a Resectable Tumor after Denosumab Neoadjuvant in a Large Dorsal Giant Cells Tumor: A Case Report and a Literature Review

**DOI:** 10.3390/curroncol30100675

**Published:** 2023-10-21

**Authors:** María Sereno, Silvia Roa Franco, Laura de la Reina, José Luis Campo-Cañaveral de la Cruz, Marta Muñoz de Legaría, Enrique Casado Saénz

**Affiliations:** 1 Medical Oncology Department, Infanta Sofía University Hospital, Europe Avenue 32, San Sebastián de los Reyes, 28702 Madrid, Spain; silvia.roa@salud.madrid.org (S.R.F.); ecasado@salud.madrid.org (E.C.S.); 2 European University of Madrid, Medicine Departtment, Calle Tajo 1, Villaviciosa de Odón, 28745 Madrid, Spain; campo-canaveral.delacruz@salud.madrid.org; 3 FIIB HUIS HHEN, 28703 Madrid, Spain; 4 Precision Nutrition and Cancer Program, Clinical Oncology Group, IMDEA Food Institute, CEI UAM, CSIC, 28049 Madrid, Spain; 5 Neurosurgeon, Neurosurgery Department, Puerta de Hierro University Hospital, C. Joaquín Rodrigo, 1, Majadahonda, 28222 Madrid, Spain; ldereina@salud.madrid.org (L.d.l.R.); marta.munoz@salud.madrid.org (M.M.d.L.); 6 Thoracic Surgery Department, Puerta de Hierro University Hospital, C. Joaquín Rodrigo, 1, Majadahonda, 28222 Madrid, Spain; 7 Pathology Department, Infanta Sofía University Hospital, Europe Avenue 32, San Sebastián de los Reyes, 28702 Madrid, Spain

**Keywords:** bone giant cell tumor, denosumab, neoadjuvant

## Abstract

Giant cell tumors of bone are a rare entity, usually occurring in young patients and characteristically arising in the long bones. The spinal location is rare and usually presents with pain and/or neurological symptoms. The treatment of choice is surgery. Treatment with Denosumab, a bisphosphonate inhibitor of RANK-L, which is highly expressed in these tumors, has shown extensive activity in unresectable patients or those undergoing incomplete surgery. Preoperative treatment with this drug is gaining increasing interest, as its high potency in tumor reduction in this subtype of neoplasm has allowed resectability in selected patients. We present the case of a young patient with a large spinal tumor who, after neoadjuvant Denosumab, underwent complete en bloc surgery with clean margins and a great pathological response.

## 1. Introduction

Bone giant cell tumors (BGCTs) comprise 5% of all bone tumors and they classically are benign tumors but with local aggressiveness; however, up to 2–3% of cases produce metastases, mainly in the pulmonary location and with a less aggressive behavior than pulmonary metastases from other types of malignant tumors such as sarcomas [1]. They are more common in females and usually occur in the third to fourth decade. The most frequent location is in the long bones, and involvement of the spine (mainly vertebral bodies, pelvis, and sacrum) represents 1–5%. Patients with tumors in the axial skeleton (SGTC) may present with neurologic signs and symptoms [2]. The risk factors related to the development of this type of tumor are unknown, although an increased incidence with respect to the general population has been seen in those patients with Paget’s disease, and several authors have described familial aggregations with Paget’s and BGCTs [3]. With respect to the pathological characteristics, BGCTs are characterized by being very vascularized tumors with cystic degeneration, hemosiderin deposition, and hemorrhage, mainly in large tumors. BGCTs typically comprise RANK (Receptor Activator of Nuclear Factor κ B)-positive circular mononuclear cells, “reactive” RANK-positive multinucleated giant cells, “tumoral” cellular spindled RANK-L (Receptor Activator of Nuclear Factor κ B Ligand)-positive stromal-like tumor cells as well as areas of osteoid matrix and woven bone [4]. Based on histology, local extension, and radiological findings, a classification has been established for this type of tumor [5]. This scale includes three grades: Grade I—intraosseous lesions with well-marginated borders and an intact cortex; Grade II—more extensive intraosseous lesions having a thin cortex without loss of cortical continuity (IIA—without pathologic fracture, and IIB—with pathologic fracture); Grade III—extra-osseous lesions that break through the cortex and extend into soft tissue.

The main treatment option for BGCTs is local therapy, ranging from en bloc resection to curettage. En bloc resection is the main treatment and can be curative if an adequate margin resection is possible. In the absence of such a complete resection, recurrence rates of 55% have been reported for primary tumors [6,7]. 

Radiation therapy (RT) is a treatment option when surgery is not possible. It produces a reduction in tumor size and a benefit in symptomatic control with acceptable disease control rates (58% of patients with no progression at 5 years). However, it has been shown that the administration of RT in these patients is associated with an increase in the malignant potential transformation of the primary lesion, sometimes as high as 10% [8].

Palliative embolization is another local treatment option when neither of the other two options is feasible for symptomatic control [9]. Those patients who are radiotherapy resistant could be treated with the high-intensity focused ultrasound technique. Several studies [10,11] showed its ability to achieve effective pain control within a couple of weeks and in some cases, HIFU also seems to be effective in tumor control with a high rate of partial response recorded [12,13].

**Figure 1:**
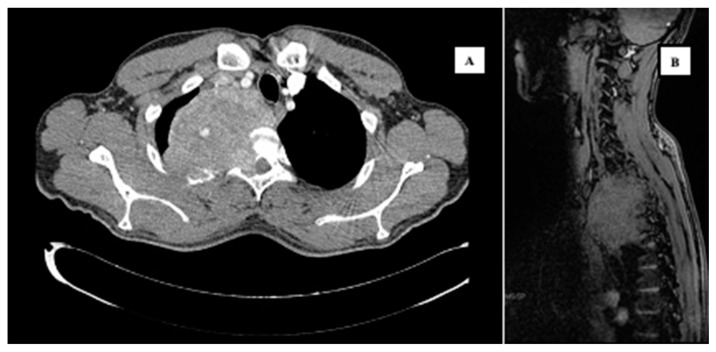
Images of the mass (July 2022) by CT with contrast (**A**) and by MRI (**B**), showing infiltration in the ipsilateral posterior third costal arch and in the right T2 and T3 vertebral hemibody, as well as occupation of the conjunctival foramina and right lateral epidural grase in T2–T3 and T3–T4, without invading thecal sac or medullary cord (**B**).

Bifosfonates have been tested in GTCBs with local activity. Denosumab, a monoclonal antibody against RANK-L, is the only drug approved by the US Food and Drug Administration (FDA) and the European Medicines Agency (EMA) for unresectable BGCTs [10] (Figure 1). However, recently, adjuvant (AD) and neoadjuvant (NA) scenarios have been tested in these patients, especially in patients with affected resection margins or in those extensive tumors in which entry surgery would be associated with great morbidity and, therefore, an effective preoperative treatment with reduction in tumor size and invasion of neighboring structures could facilitate an en bloc or curative resection [9]. 

We present the case of a patient diagnosed with a SGCT with double vertebral involvement (T2–T3) with a wide costal and pulmonary extension who, after a very good response to NA denosumab, successfully underwent en bloc resection with free margins and an excellent pathologic response. We present the following case in accordance with the CARE reporting checklist.

## 2. Case Report

A 39-year-old man presented to the emergency department with a persistent cough that had been bothering him for 2–3 months. He did not experience any other respiratory symptoms. He mentioned having mild pain between his shoulder blades, but there were no other accompanying neurological symptoms. He denied having heartburn or any other gastrointestinal symptoms. Additionally, he had lost 2–3 kg of weight, had recurring fevers, and occasional night sweats. He did not notice any swollen lymph nodes when he examined himself. The patient had no significant past medical history and had not been exposed to toxins or received any regular treatment. In his family history, his father died of stomach cancer and his maternal grandmother had colon cancer, both before the age of 60. Upon examination, no notable findings were observed during auscultation or palpation. Urine and blood tests did not show any abnormalities. A chest X-ray revealed a large mass outside the lungs in the right pulmonary apex, which raised suspicion of malignancy. Consequently, the patient was admitted for further investigation. Tumor markers, including β-HCG, α-fetoprotein, β2-microglobulin, and proteinogram, were all within normal ranges.

**Figure 2:**
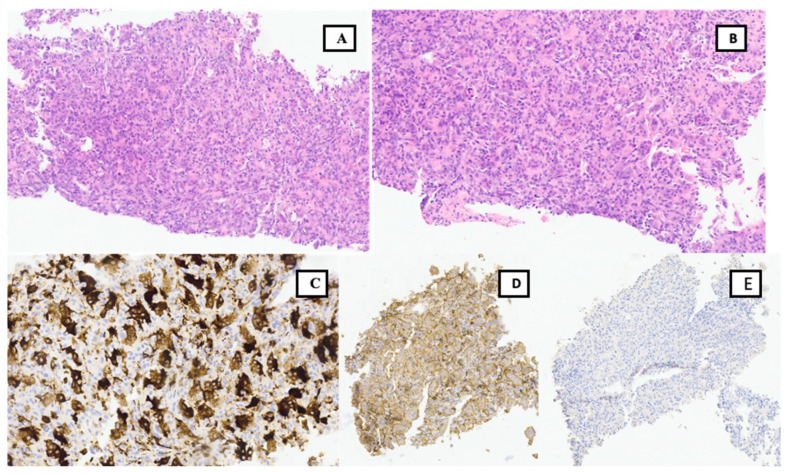
Pathological study of BAG: Haematoxylin-eosin staining 20×, different magnifications. (**A**,**B**). Multitude of multinucleated giant cells, without atypical features. Positivity for CD 163, CD 68 (**C**) and CD 45 (×40) (**D**). Negative pattern for pancytokeratin marker (**E**), TTF-1, CD34, S100, CEA, Ki 67, p40, and AML.

**Figure 3:**
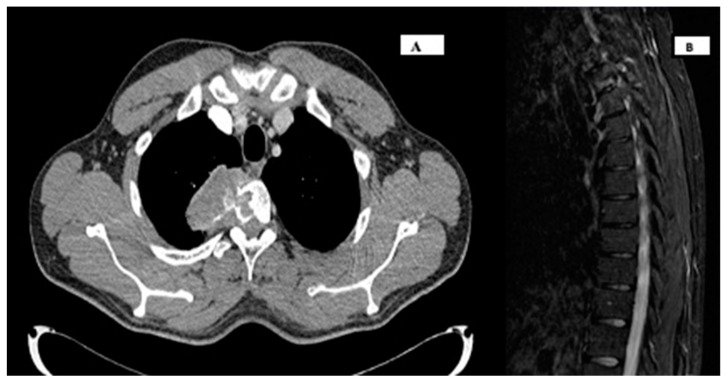
A/B. Re-evaluation studies after neoadjuvant treatment (January 2023). (**A**) Thoracic CT scan showing response to treatment, with a significant decrease in the size of the right posterior costovertebral mediastinal mass, with diameters currently approximately 3.5 × 5.9 × 4.3 cm (AP × T × CC) and similar invasion/affectation of the right side of the T2 and T3 vertebrae, respecting the contiguous T1 and T4 vertebrae. (**B**) MRI of the dorsal spine also showing a marked decrease in the soft tissue mass associated with lytic lesion in the right lateral mass of the T3 vertebral body and a decrease in the dimensions of the spinal canal on the right side without obliteration of the exit foramina of the T2 and T3 roots.

Once the presence of a paramediastinal mass measuring 8.5 × 10 × 9 cm (AP × T × CC) was confirmed through thoracic CT, which suggested a possible neurogenic origin, samples (plasma and urine) were collected to screen for paraganglioma. Since metanephrines and catecholamines were within normal ranges, it seemed more plausible to consider a primary bone tumor. The study was further complemented with an MRI of the thoracic spine and a PET CT scan, revealing infiltration in a single rib and multiple vertebrae at the T2 and T3 levels. However, spinal cord invasion was ruled out, including the right margin of the T2–T4 vertebral canal (Figure 1). A biopsy was performed on the lesion, confirming a diagnosis of a stage III giant cell tumor with extraosseous extension (Figure 2). The case was presented to the multidisciplinary tumor committee, and after consultation with the Thoracic Surgery and Neurosurgery, it was decided to initiate neoadjuvant treatment with Denosumab to optimize the tumor size and improve resectability options, given the inability to achieve an R0 resection. Due to the patient’s excellent tolerance and positive response, without any new clinical manifestations, it was decided to continue Denosumab for 6 months, resulting in a remarkable locoregional response (Figure 3A,B) before proceeding with subsequent surgical intervention.

**Figure 4:**
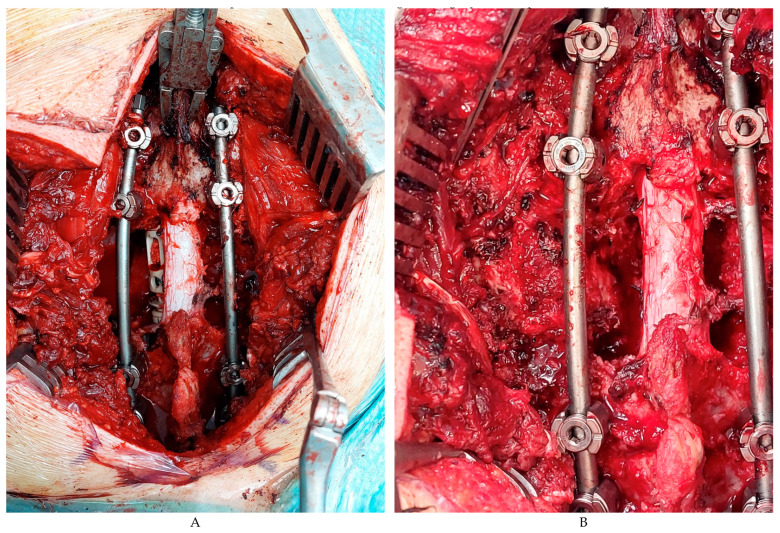
(**A**,**B**) Intraoperative images of the result of T2–T3 vertebrectomies with expandable PEEK radiolucent vertebral body replacement (XRL^®^, Johnson & Johnson) filled with a synthetic bone graft substitute (Vitoss^®^, Stryker) was implanted.

The patient underwent a posterior approach, with intraoperative neurophysiological monitoring. We made a section of the proximal part of the second, third, and fourth right ribs. Pedicles screws were inserted in C7, T1, T4, and T5 (Expedium^®^ system, Johnson & Johnson, Mentor Medical Systems Ibérica, Madrid, Spain) which were navigated and O-arm controlled. A more lateral periscapular incision was made to expose the lateral aspects of the second, third, and fourth ribs, which were transected 5 cm from the spine. The neurovascular structures at each level were ligated. Additionally, due to the potential involvement of the lung parenchyma, a wedge resection was performed. Finally, the anterior vertebral ligament was sectioned to extract the final specimen. Since the chest wall resection was small and located posteriorly and superiorly, mesh reconstruction was not deemed necessary. A chest tube was placed at the end of the procedure. Once the spinal was stabilized, T2–T3 vertebrectomies were performed and the right paramediastinal mass was removed with a small lung piece. Finally, an expandable PEEK radiolucent vertebral body replacement (XRL^®^, Johnson & Johnson) filled with a synthetic bone graft substitute (Vitoss^®^, Stryker Howard Ct, Clearwater, FL, USA) was implanted. The spinal cord function during all surgery was kept intact (Figure 4A,B).

**Figure 5:**
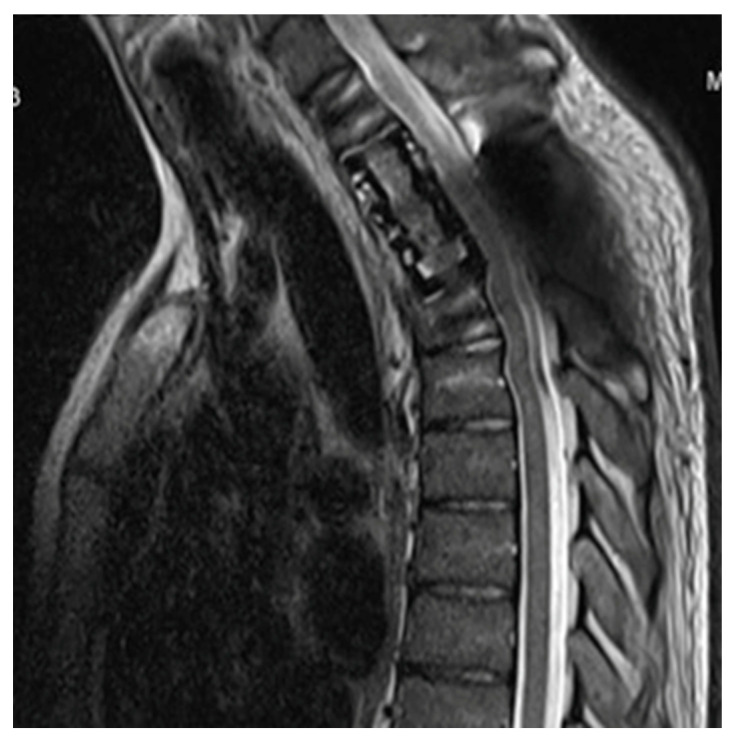
T1-weighted MR image two months after surgery showing post-surgical changes with no evidence of recurrence.

The surgical specimen, which included three costal segments, part of the vertebral bodies (T2 and T3), a lung segment, and adjacent skeletal muscle, showed pathological changes in response to the giant cell tumor after Denosumab treatment. The tumor extended into the soft tissues and had a maximum dimension of 5.5 cm. During the vertebra resection, fragments of cancellous bone with a giant cell tumor after Denosumab were observed, along with intense reactive bone at the periphery. Fragments of unaffected hyaline cartilage, fibrocartilage, and skeletal muscle were also found without giant tumor cells. The patient was discharged without any postoperative complications after undergoing T2–T3 corporectomy. Postoperative follow-up involves a dorsal MRI every 3 months. The latest CT scan shows post-surgical changes with no signs of persistence or recurrence of his oncological pathology (Figure 5).

## 3. Discussion

BGCTs are typically located in the long bones, so their localization in the spine is extremely rare. In vertebral locations, especially in large tumors, the presence of pain and neurological symptoms usually occurs in up to 70% of cases. It is noteworthy that in our case, the patient had very few symptoms and the examination revealed no neurological focality, neither sensory nor motor. The absence of symptomatology in this case probably conditioned the late diagnosis with a large lesion and a large local extension [11]. 

As previously mentioned, en bloc resection is the treatment of choice; however, this surgery is subject to non-negligible morbidity and functional complications and the possibility of local recurrence despite its radicality, especially if the resection margins, are not adequate. Depending on the experience of the centers and the cases, this type of surgery has a mortality rate of around 15%. In fact, the spinal location has a high percentage of unresectability and is associated with a high risk of pathological fractures [12]. The intrathoracic and mediastinal extension as well as the location of our patient’s lesion (involvement of T2 and T3, with doubts of punctual extension to T4 judging by the imaging tests) made primary resection complex, implying surgery associated with great morbidity and little guarantee of obtaining free margins at front. However, although radiotherapy can be an alternative to surgery as previously described, with an acceptable rate of local control, it can be used as an alternative to surgery [13]. In our patient, with a potentially resectable tumor after a potential partial reduction with systemic treatment, it seemed to us to be an option to consider in the case of affected margins after an attempt at en bloc resection following local response to systemic treatment or as an alternative in case of local recurrence.

**Figure 6:**
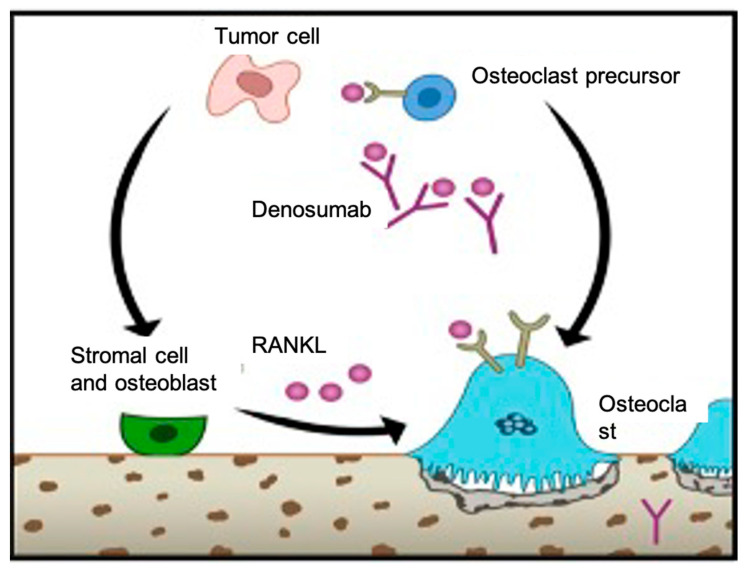
Pathophysiology of RANKL. We know that tumor giant cells are activated osteoclasts through an indirect effect on osteoblasts and stromal cells by the presence of a stimulatory factor: RANKL. Denosumab specifically binds to RANKL, extinguishing the RANK positivity of GCT cells, preventing their osteolytic functionality [6].

Prior to Denosumab, no medical treatment based on chemotherapy or other bisphosphonates had demonstrated efficacy in this type of tumor. Its physiopathology, with a high intratumoral target concentration (RANK-L), makes it particularly susceptible to the activity of a targeted therapy against these ligands, reaching high intratumoral concentrations and achieving a high antitumor activity [14] (Figure 6). Several authors have published experiences and case series with the administration of Denosumab in spinal BGCTs in different perioperative contexts or as the only treatment in patients considered unresectable (in different locations, not only the spine). Table 1 shows different experiences or studies from different authors with Denosumab in neoadjuvant settings in patients with SGCTs. Thomas D et al. presented one of the first series with 37 patients diagnosed with recurrent or unresectable BGCTs treated with subcutaneous Denosumab 120 mg monthly (every 28 days), with loading doses on days 8 and 15 of month 1. They observed a tumor response of 100% biopsies and 66% radiological responses. Mild adverse were described: extremity and back pain and headache in less than 20% of all participants [15]. 

Another individual experience of a SGCT in T9 has been reported. In this case, the patient was 18 years old and received neoadjuvant treatment for 9 months until maximum response with excellent clinical and analytical tolerance, achieving a complete response in the surgical specimen after vertebral curettage and adjuvant treatment with Denosumab until completing one year of treatment [16]. 

Continuing with studies on the role of neoadjuvant Denosumab, Rutkowski P et al. published a phase II clinical trial focused on the degree of response of Denosumab in the neoadjuvant setting in patients with advanced BGCTs. They found that around 48% of 222 patients had no surgery or a less morbid procedure because of a local dramatic response. A total of 116 patients underwent surgery developing local recurrence in 17 patients (15%). The median duration to recurrence was 13.6 months, postoperatively [17]. 

Other authors such as Chawla et al. recently published a phase II study with 282 patients with BGCTs in different locations that included three cohorts of adult patients (between 12% in cohort 1 and 27% in cohort 3 of spine location): Cohort 1 of patients with unresectable disease; Cohort 2 with patients with potentially resectable tumors; and Cohort 3 derived from patients from a previous study with Denosumab. All patients received Denosumab every 4 weeks at usual doses (120 mg/4 weeks) with the loading dose at days +8 and +15. The primary objective was a safety analysis in relation to ionic alterations and secondary objectives were for Cohort 1 SLE and for the Cohort 2 percentage of patients who had not undergone surgery at 6 months. In this study, Denosumab was administered until progression or intolerance in unresectable cases and in patients who underwent surgery. Six adjuvant doses were subsequently administered along with vitamin D supplementation. The most frequent adverse effects described with this treatment were arthralgias (20%), headache (18%), and nausea (17%). Regarding the percentage of surgeries in Cohort 2 (101 patients included), there were 44 major resections including joint resections and prosthesis placement, and 37 en bloc resections, with some marginal excision and curettage. Complete response rates ranged from 5% in Cohort 1 to 18% in Cohort 2 with a large symptomatic benefit in both cohorts, superior in those patients undergoing other surgery (Cohort 2) [9]. 

Our patient, similar to those included in Cohort 2 of the study by Chawla S et al., had a potentially resectable tumor with a very good response to Denosumab allowing an en bloc resection without complications and achieving a considerable functional benefit. As mentioned above, the patient also showed excellent tolerance to bisphosphonate with R0 resection and a complete pathological response as we have previously mentioned.

Another case series published in the literature that analyzed, in the same way, the role of neoadjuvant Denosumab in patients with spinal BGCTs was collected by Dubory et al. They analyzed the evolution of nine operated patients, four of them having received neoadjuvant treatment with Denosumab. Surgical procedures included six osteosyntheses, one en bloc resection, and 4 curettages. The median duration of time with Denosumab was 12.9 months and 6 months in the cases that received preoperative treatment with a pathologic result showing the absence of giant cells and less than 10% of viable tumor tissue [18]. The duration of preoperative treatment was similar to our case, close to 6 months where the radiological response obtained allowed the surgeons a safe and radical approach in our patient. There is no answer on the duration of NA treatment with this drug. Most authors suggest an estimated duration of between 3 and 6 months, with an initial loading dose consisting of 3 weekly administrations of 120 mg subcutaneous at the beginning, continuing with the standard monthly regimen thereafter. 

Hindiskere S et al. published that short treatments of no more than 3 months have similar results in terms of radiological and pathological responses compared to longer treatments (6 months) with lower cost and toxicity [19]. In our patient, we observed a progressive decrease in the size of the initial lesion in the intermediate reevaluations performed with CT scans during neoadjuvant treatment with Denosumab, observing a greater response after each dose was administered, so it was decided to maintain a prolonged part of treatment reached 6 months of preoperative therapy. Several authors have described more surgical complications in those patients who received prolonged neoadjuvant treatment (>9 months) associated with a higher risk of vertebral fracture and new osseous tumor matrix and thickened cortical bone developed with Denosumab treatment do not allow the surgeon to delineate the true extent of the tumor and might increase the risk of local recurrence after intralesional therapy [20]. 

However, despite these potential complications from prolonged use of Denosumab, administration of NA Denosumab has reduced bleeding complications during the surgical procedure. Yang et al. compared two groups of patients with sacral GCT: a neoadjuvant Denosumab arm and an untreated control group, finding less bleeding and need for transfusions as well as a higher rate of local responses in the Denosumab arm [21]. Other authors have published combinations with antiangiogenic drugs, with encouraging results. For example, a study comparing the association between Levantinib and Denosumab vs. Denosumab alone was found to be more effective in terms of disease control [22]. Further studies are needed to consolidate this strategy as standard systemic treatment in this subgroup of patients. Another potential complication associated with Denosumab treatment in the perioperative setting is the lack of benefit in reducing the risk of local recurrence in patients who, after treatment with neoadjuvant Denosumab, undergo intralesional curettage. Traub et al. published a local recurrence rate of up to 17% comparable to studies with curettage without neoadjuvant treatment [23]. Errani et al. demonstrated a worse outcome in patients treated with Denosumab and curettage versus curettage alone in a series of patients with GCTBs (60% vs. 16%). However, this was not seen in patients who underwent radical en bloc resection after neoadjuvant treatment [24,25,26]. 

There is no evidence that, in this case, the administration of adjuvant Denosumab has a clear impact on the prevention of recurrences in the absence of residual disease. In addition, several studies describe the response to Denosumab in patients previously treated with Denosumab and with an initial response [9].

However, not all patients respond equally to Denosumab. Epigenetic modifications of histones, specifically in the H3 histone family 3A (H3F3A) gene, which are present in over 90 percent of GCTBs, may be driving tumorigenesis.

Recently, a mutation has been identified in the H3F3A gene (G34W) and to a lesser extent (G34L) that has been associated with resistance to Denosumab in this type of tumor [27]. 

After surgery, according to pathological results, we have decided on an observation protocol. Studies support doing nothing after surgery if there is a complete response [26,28,29].

## 4. Conclusions

Our case report illustrates once again the role of Denosumab in different areas in the preoperative setting for SGCTs with extensive locoregional involvement. It has allowed a significant reduction in tumor burden transforming an unresectable tumor into a resectable one. On the other hand, it has facilitated surgery reducing hemorrhagic complications and finally, it has allowed en bloc resection with free borders optimizing local control of this type of lesions. Tolerance to treatment has been excellent and the pathological response obtained has been complete.

## Figures and Tables

**Table 1:** This table shows a brief of different studies/experiences with Neoadjuvant Denosumab in patients with spine tumors.

Author’s	Study	N of Patients	Location	Resectability	Treatment	Pathology
Thomas et al. [15]	Serial cases	37	Spine and other locations	Unresectable, recurrent disease	Denosumab: loading dose and monthly dose	100% biopsies and 66% radiological responses
Goldschlager T [16]	Case report	1	T9	Potential resectable	Neoadjuvant and adjuvant Denosumab 9 months	Complete response
Rutkowski P [17]	Phase II	222	Spine and other locations	116 patients	Neoadjuvant Denosumab	99 complete response 17 recurrence (median TTR 13.6 m)
Chawla et al. [9]	Phase II	282	Spine and other locations	Cohort 1: resectable Cohort 2: unresectable	Cohort 1: neoadjuvant Denosumab and 6 months adjuvant Denosumab Cohort 2: Denosumab till progression	Cohort 1: 5% complete response Cohort 2: radiological 18% complete response
Dubory et al. [18]	Serial cases	9	Spine and other locations	Resectable or potentially resectable	Neoadjuvant Denosumab	10% complete response

## Data Availability

The datasets used and/or analyzed during the current study are available from the corresponding author upon reasonable request.

## Abbreviations

BGCT	Bone giant cell tumors
SGTC	Spine giant cell tumors
RANK	Receptor Activator of Nuclear Factor κ B
RANK-L	Receptor Activator of Nuclear Factor κ B Ligand
RT	Radiation therapy
AD	Adjuvant
NA	Neoadjuvant
FDA	US Food and Drug Administration
EMA	European Medicines Agency
CT	Computerized tomography
PET CT	Positron emission tomography computerized tomography
